# Dual Impact of Estradiol Fluctuations in the Final Three Days on IVF/ICSI Outcomes: A Retrospective Cohort Analysis

**DOI:** 10.5935/1518-0557.20250038

**Published:** 2025

**Authors:** Caixia Xiao, Miaochen Long, Qiulian Xu, Ling Bu, Yawen Yu, Xueyu Lu, Changmin Niu, Tongmin Xue

**Affiliations:** 1 School of Nursing, Faculty of Medicine, Yangzhou University, Yangzhou, 225009, PR China; 2 Yangzhou University Affiliated Northern Jiangsu People’s Hospital. Yangzhou, Jiangsu Province, China

**Keywords:** estradiol dynamics, progesterone fluctuation, ovarian stimulation protocol, IVF/ICSI outcomes, individualized hormone management

## Abstract

**Objective:**

How do dynamic changes in serum estradiol (E2) and progesterone (P) concentrations during controlled ovarian hyperstimulation (COS) affect oocyte retrieval and embryo outcomes? What clinical factors drive estradiol elevation in the final three days of stimulation?.

**Methods:**

This single-center retrospective study included 486 first-cycle patients with normal ovarian function undergoing a long protocol in early follicular phase, with fresh embryo transfer. Serum E2 and P concentrations were measured at four time points: the gonadotropin initiation day, four days post-initiation, two days before the hCG trigger, and the day of hCG administration, with different stages, grouped patients by median hormone changes into high-growth and low-growth. Primary and secondary endpoints were ovarian stimulation and pregnancy outcomes, respectively. logistic regressions identified factors associated with estradiol elevation during the last three days of stimulation.

**Results:**

High-growth groups had higher oocyte retrieval rates but lower transferable embryo rates. Multivariate analysis identified higher AFCs and mid-cycle estradiol increase as a protective factor (*p*<0.01). Conversely, higher BMI, higher initial Gn dose (>300 IU) and prolonged stimulation duration were linked to poorer outcomes (*p*<0.01).

**Conclusions:**

Dynamic E2/P monitoring provides actionable insights for COS management. Elevated E2 in the late stimulation phase, driven by AFC and mid-cycle hormonal surge, correlates with oocyte yield but requires caution due to reduced embryo utilization. Clinically, excessive gonadotropin dosing and prolonged stimulation should be avoided to optimize outcomes. These findings support personalized hormone adjustment protocols to balance efficacy and safety in ART cycles.

## INTRODUCTION

In assisted reproductive technology (ART), in vitro fertilization (IVF) and intracytoplasmic sperm injection (ICSI) remain the primary techniques for treating infertility. Both procedures rely on serum hormone assays to assess ovarian reserve and monitor the response to controlled ovarian stimulation (COS) (Kwan *et al*., 2021). Key hormonal markers include anti-Müllerian hormone (AMH), follicle-stimulating hormone (FSH), luteinizing hormone (LH), estradiol (E2), and progesterone (P). These measurements inform gonadotropin dosing and guide treatment protocols (Al-Inany *et al.*, 2016).

Recent advances in gonadotropin-releasing hormone analogues (GnRHa) and gonadotropins (Gn) have improved follicular recruitment by modulating the hypothalamic-pituitary-gonadal axis (Yutian *et al*., 2023). These treatments coincide with hyperproliferative changes in serum hormone concentrations (Maggi *et al*., 2016), and serum E2 and P concentrations are among the most frequently monitored markers (Fauser *et al*., 2002). Studies have demonstrated that elevated E2 levels during COS are closely associated with ovarian hyperstimulation syndrome (OHSS) onset (Cheng *et al*., 2020; Sachs-Guedj *et al*., 2023). Additionally, increased P levels - whether isolated or present on the trigger day - correlate with adverse outcomes, including reduced clinical pregnancy and live birth rates, even in patients exhibiting favorable ovarian reserve and response (Venetis *et al*., 2013). A comprehensive meta-analysis involving over 60,000 cycles concluded that serum P levels above 0.8 ng/mL on the day of hCG administration are linked to a higher risk of pregnancy loss in fresh IVF cycles (Oktem *et al*., 2019).

The impact of estradiol and progesterone fluctuations during COS has garnered significant attention in reproductive medicine, establishing this as a key research focus. Research suggests that patients who experience a decrease in estradiol levels before the trigger day tend to have fewer retrieved oocytes (Ling & Yao-fang, 2023; Zhu *et al*., 2024). However, the predictive value of estradiol levels on the trigger day for pregnancy or birth outcomes remains inconclusive (Lersten *et al*., 2024). While most studies focus on E2 and P levels at COS initiation or on the day of hCG administration, our study investigates dynamic changes in these hormones across three COS stages and four time points. We aim to identify factors contributing to the rise in estradiol during the final three days of the cycle, with the goal of improving hormone monitoring and clinical decision-making.

## MATERIALS AND METHODS

### Study design and Study population

This retrospective study analyzed clinical data from patients who underwent IVF/ICSI treatment at a university-affiliated reproductive medicine center between January 2021 and December 2023. Inclusion criteria were: first stimulation cycle, use of a long protocol in early follicular phase, fresh embryo transfer, and normal ovarian function (AMH >1.1 ng/mL, AFC <20) (Broer *et al*., 2014; Das *et al*., 2024; La Marca & Sunkara, 2014). Exclusion criteria included unilateral oophorectomy, incomplete cycle data, use of donor gametes, chromosomal abnormalities in either partner, and ovarian stimulation duration ≤9 days.

A total of 486 patients met the criteria and were included in the analysis. Serum estradiol (E2) and progesterone (P) levels were assessed at four time points during controlled ovarian stimulation (COS): gonadotropin initiation day (Gn day), day 4 of stimulation (Gn+4), two days before hCG trigger (HCG-2), and hCG administration day (HCG day). Hormonal changes were analyzed across three COS stages: Stage 1 (Gn to Gn+4), Stage 2 (Gn+4 to HCG-2), and Stage 3 (HCG-2 to HCG). Due to undetectable P levels at Gn day, only two stages were used for progesterone analysis. Patients were categorized into high-growth and low-growth groups based on median hormone level changes at each stage.

### Study procedures

From 2020 to 2023, five 2-mL blood samples were collected from each participant at defined time points: baseline, Gn day, Gn+4, HCG-2, and HCG day. Baseline samples measured FSH, LH, E2, testosterone, and AMH. The four subsequent samples were tested for serum LH, FSH, E2, and P. All blood samples were drawn between 7:00 and 9:00 AM to minimize circadian variability. Clinical and demographic data were recorded, including age, BMI, infertility type and duration, etiology, baseline hormone levels, uterine volume, and endometrial thickness. Ovarian stimulation parameters-starting dose, cumulative dose, and duration-were also documented.

To suppress endogenous LH and FSH secretion and prevent premature ovulation, long-acting GnRH agonists (e.g., triptorelin or leuprolide) were administered on cycle days 2-3. Down-regulation typically required ~28 days. Upon confirmation, ovarian stimulation was initiated with recombinant FSH (rFSH) or human menopausal gonadotropin (hMG). The starting dose was individualized based on age, BMI, basal FSH, AFC, and AMH levels. Follicular development was monitored via transvaginal ultrasound and serial hormone measurements (E2, P, FSH, LH). Gonadotropin doses were adjusted accordingly. Final oocyte maturation was triggered with hCG or GnRH agonist when dominant follicles reached 16-20 mm in diameter. Oocyte retrieval was scheduled 34-36 hours later (ESHRE Guideline Group on Ovarian Stimulation *et al*., 2020; Ovarian Stimulation TEGGO *et al*., 2020; Yun *et al*., 2023). Standard luteal phase support was provided prior to fresh embryo transfer. Embryos were selected based on morphological criteria, and transfer was performed under ultrasound guidance to optimize implantation (Khattak & Amorim, 2022).

### Endpoint

Primary outcomes included: Oocyte retrieval rate; Metaphase II (MII) oocyte rate; 2PN fertilization rate; 2PN cleavage rate; High-quality embryo rate; Usable embryo rate; For IVF cycles, 2PN fertilization rate = (2PN oocytes on Day 1 / total oocytes retrieved) × 100%. For ICSI, 2PN fertilization rate = (2PN oocytes / MII oocytes injected) × 100%. Cleavage rate = (cleaved fertilized embryos / total fertilized embryos) × 100%. Usable and high-quality embryo rates were calculated similarly. Clinical pregnancy was defined by the presence of a gestational sac with fetal heartbeat on ultrasound. Live birth was defined as delivery of a live infant after 24 weeks of gestation (Gosden *et al*., 2003; Khattak & Amorim, 2022).

### Statistical analysis

Data were analyzed using SPSS v26. Continuous variables are presented as medians with interquartile ranges (IQRs); categorical variables as frequencies and percentages. Group comparisons were performed using chi-square or Mann-Whitney U tests, as appropriate. Univariate and multivariate logistic regression, as well as multiple linear regression, were used to evaluate the effects of E2 and P at different stages on IVF outcomes and to identify factors associated with rising E2 levels in the final three days of stimulation.

## RESULTS

### Patient population

This study included 486 patients who met the inclusion criteria. Table 1 summarizes demographic characteristics and pregnancy outcomes; Table 2 details baseline variables, hormone levels, and ovarian stimulation results. Most patients (70.99%) were aged 22-32, with pelvic or tubal factors being the most common infertility causes.

**Table 1:** The demographic details and pregnancy outcomes.

Characteristic and outcomes	group	F	%
Type	Primary infertility Secondary infertility	286 200	58.85 41.15
Age	22-32 33-43	345 141	70.99 29.01
BMI	Underweight Normal Overweight Obese	18 277 155 36	3.70 57.00 31.89 7.41
Reason	Pelvic and tubal factors Decreased ovarian function Disturbance of ovulation Endometriosis unknown Cause Male partner factor	171 56 48 47 86 78	35.19 11.52 9.88 9.67 17.70 16.05
Source of embryo	IVF ICSI Rescue ICSI	374.00 79.00 33.00	76.96 16.26 6.79
Biochemical pregnancy	Yes No	311 175	63.99 36.01
Clinical pregnancy	Yes No	280 206	57.61 42.39
Live birth	Yes No	211 275	43.42 56.58

**Table 2:** The Basal levels and ovulation induction outcomes.

Basal levels and ovulation induction outcomes	M	P25-P75
Year	2.59	1.98-4.0
AFC	11.00	8-14
AMH(ng/ml)	2.68	1.86-3.88
FSH(IU/L)	6.44	5.49-7.51
LH(IUL)	4.86	3.48-6.38
E2(pmol/L)	120.00	98.85-152
PRL(mIU/L)	17.98	13.58-23.5
T(nmol/L)	0.81	0.58-1.06
Endometrium	6.00	4.48-7.7
Ovaries volume	3733.57	2684.23-5217.97
E2 change in stage 1(pmol/L)	218.30	109.35-393.85
E2 change in stage 2(pmol/L)	2404.00	1422.53-3539.38
E2 change in stage 3(pmol/L)	2577.50	1566.25-3801.75
P change in stage 2(pmol/L)	270.00	0-572.5
P change in stage 3(pmol/L)	423.00	140.75-832.5
Oocytes Retrieved Rate (%)	71.43	57.14-87.50
High-Quality Oocytes Rate(%)	100.00	85.71-100.00
2PNFertilized Oocytes Rate (%)	75.00	60.00-90.00
2PN Cleavage Stage Embryos Rate(%)	100.00	100.00-100.00
Transferable Embryos Rate (%)	100.00	83.33-100.00
High-Quality Embryos Rate(%)	66.67	40.00-89.17

**Figure 1:**
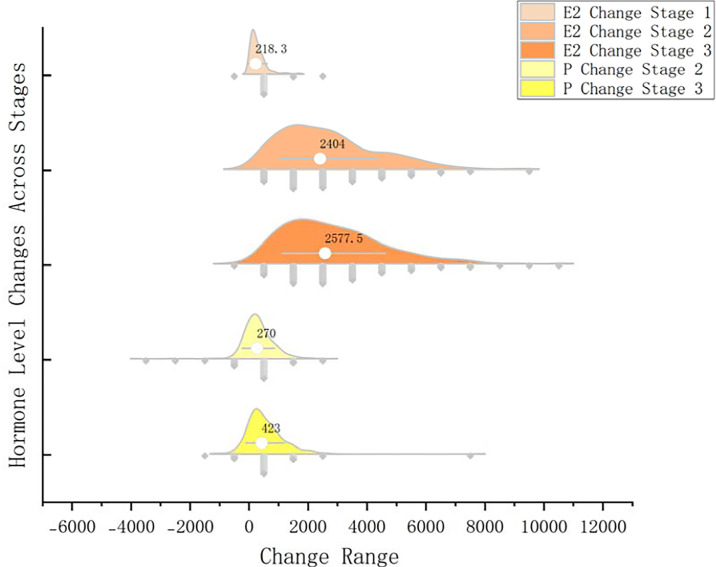
Hormone fluctuations in different stages.

Median serum E2 increments across the three COS stages were 218.3, 2392.0, and 2577.5 pmol/L, showing significant rises throughout. Median P values in stages 2 and 3 were 270.0 and 423.0 pmol/L, respectively, with a significant increase between stages (*p*<0.05). Hormone fluctuations are illustrated in Figure 1.

### Primary and secondary outcome measures

**Figure 2:**
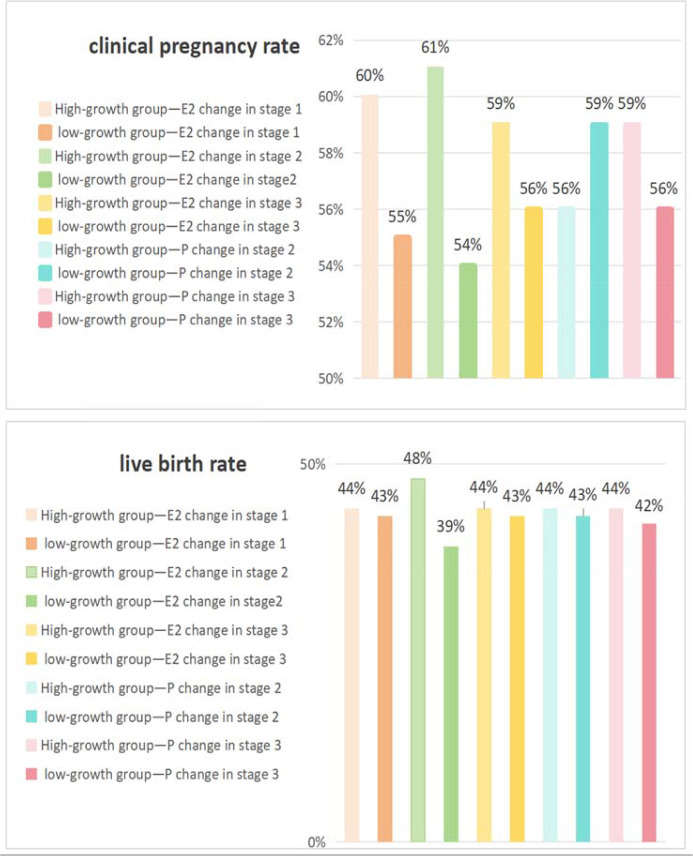
Clinical pregnancy rate and live birth rate of different stages’ patients.

Using nonparametric methods as presented in Table 3, patients were grouped into highand low-growth subgroups based on median E2 and P changes. The Mann-Whitney U test revealed no significant differences in key embryological outcomes during the first stage (*p*>0.05). However, during stages 2 and 3, high-growth groups showed higher oocyte retrieval rates. Unexpectedly, the transferable embryo rate was lower in the high E2 growth group. In stage 3, the low-growth E2 subgroup had a higher 2PN cleavage rate; in the P subgroup, it had a higher high-quality embryo rate. Clinical pregnancy and live birth rates did not differ significantly between groups (*p*>0.05), though CPR was generally higher in high-growth groups, except for the stage 2 P subgroup, showed in Figure 2.

**Table 3:** Outcome measures of two groups in different stages

OUT come Rate (%)	E2 change in stage 1 M(P25,P75)		Z	P
	High-growth group (N=243)	low-growth group (N=243)		
Oocytes Retrieved	75.0(59.2,88.9)	69.2(55.6,85.7)	-1.70	0.089
High-Quality Oocytes	100.0(85.7,100.0)	100.0(85.7,100.0)	-0.65	0.518
2PN Fertilized Oocytes	75.0(60.0,88.6)	77.8(60.0,100.0)	-1.08	0.281
2PN Cleavage Stage Embryos	100.0(100.0,100.0)	100.0(100.00,100.0)	-1.12	0.263
Transferable Embryos	100.0(83.3,100.0)	100.0(83.33,100.0)	-0.90	0.355
High-Quality Embryos	63.6(40.0,85.7)	66.7(42.86,100.0)	-1.38	0.168
Outcome Rate (%)	E2 change in stage 2 M(P25,P75)		Z	P
	High-growth group (N=243)	low-growth group (N=243)		
Oocytes Retrieved	76.9(61.5,90.5)	66.7(50.0,85.7)	-3.70	<0.001
High-Quality Oocytes	100.0(87.1,100.0)	100.0(85.7,100.00)	-1.83	0.068
2PN Fertilized Oocytes	75.0(60.0,88.9)	75.0(60.0,100.0)	-1.94	0.053
2PN Cleavage Stage Embryos	100.0(100.0,100.0)	100.0(100.0,100.0)	-0.60	0.550
Transferable Embryos	100.0(80.0,100.0)	100.0(88.9,100.0)	-3.37	0.001
High-Quality Embryos	66.7(40.00,85.7)	66.7(42.9,100.0)	-1.30	0.195
OUT come Rate (%)	E2 change in stage 3 M(P25,P75)		Z	P
	High-growth group (N=243)	low-growth group (N=243)		
Oocytes Retrieved	76.5(61.5,90.0)	66.7(50.0,84.6)	-3.64	<0.001
High-Quality Oocytes	100.0(85.7,100.0)	100.0(85.7,100.0)	-0.81	0.416
2PN Fertilized Oocytes	75.0(56.7,88.9)	75.0(62.5,100.0)	-0.48	0.631
2PN Cleavage Stage Embryos	100.0(100.0,100.0)	100.0(100.0,100.0)	-2.74	0.006
Transferable Embryos	100.0(80.0,100.0)	100.0(87.5,100.0)	-3.21	<0.001
High-Quality Embryos	66.7(41.4,85.7)	66.7(40.0,100.0)	-1.61	0.107
Outcome Rate(%)	P change in stage 2 M(P25,P75)		Z	P
	High-growth group (N=243)	low-growth group (N=243)		
Oocytes Retrieved	76.5(61.5,90.91)	66.67(50.0,83.3)	-4.19	<0.000
High-Quality Oocytes	100.0(86.2,100.0)	100.0(85.7,100.0)	-0.20	0.842
2PN Fertilized Oocytes	76.9(60.0,92.3)	75.0(60.0,88.9)	-0.24	0.808
2PN Cleavage Stage Embryos	100.0(100.0,100.0)	100.0(100.0,100.0)	-0.56	0.574
Transferable Embryos	100.0(80.0,100.00)	100.0(87.5,100.0)	-3.52	0.001
High-Quality Embryos	66.7(40.0,85.7)	66.7(50.0,100.0)	-1.87	0.061
Outcome Rate (%)	P change in stage 3 M(P25,P75)		Z	P
	High-growth group (N=243)	low-growth group (N=243)		
Oocytes Retrieved	75.0(62.5,90.0)	66.7(50.0,85.2)	-4.06	<0.001
High-Quality Oocytes	100.0(85.7,100.0)	100.0(85.71,100.0)	-1.10	0.271
2PN Fertilized Oocytes	75.00(60.0,90.0)	75.0(60.0,100.0)	-0.32	0.748
2PN Cleavage Stage Embryos	100.0(100.0,100.0)	100.0(100.0,100.0)	-0.17	0.868
Transferable Embryos	100.0(80.0,100.0)	100.0(86.6,100.0)	-2.51	0.010
High-Quality Embryos	62.5(38.7,85.7)	100.0(83.3,100.0)	-2.11	0.035

### Analysis of influencing factors on serum estradiol concentration in stage 3

**Figure 3:**
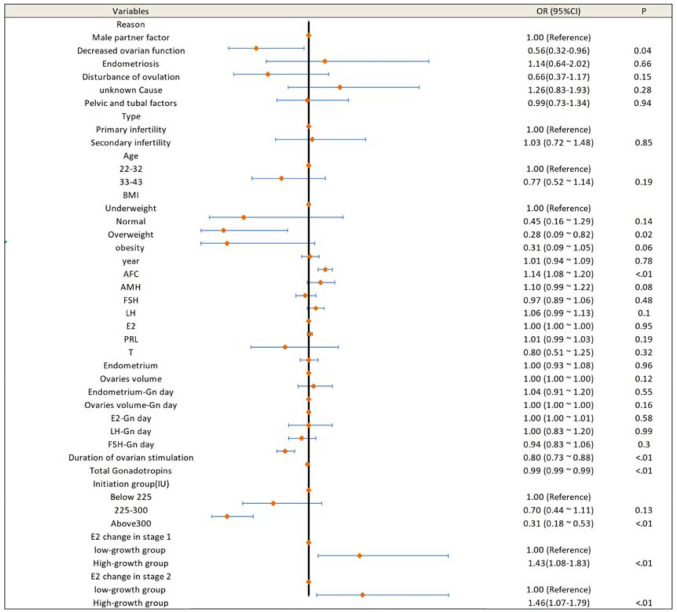
Univariate analysis outcome.

**Figure 4:**
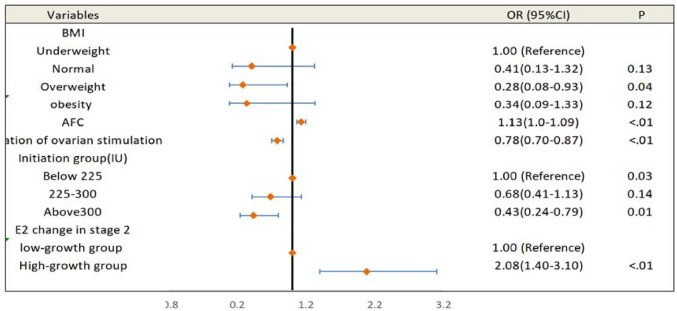
Multivariate regression outcome.

Linear regression (Table 4) identified E2 changes in stage 3 (β = 0.161, *p*<0.001) and P changes in stage 2 (β = 0.139, *p*<0.001) as positively correlated with oocyte retrieval rate. A negative correlation was observed between stage 3 E2 increase and transferable embryo rate (β = -0.150, *p*=0.003). Univariate analysis (Figure 3) showed that patients in the low E2-growth group during stage 3 had lower AFCs, higher BMI, longer stimulation, higher initial and cumulative Gn doses, and slower hormone growth in prior stages. Multivariate regression (Figure 4) identified higher AFCs (OR = 1.13, 95% CI: 1.0-1.09, *p*<0.01) and greater stage 2 E2 increase (OR = 2.08, 95% CI: 1.40-3.10, *p*<0.01) as protective factors. High starting doses (>300 IU) and longer stimulation duration were risk factors.

**Table 4:** Rate of oocytes retrieved and transferable embryos.

CHARACTERISTICS	Rate of Oocytes Retrieved		P
	B	t	
E2 change in stage 2	0.097	1.971	0.052
E2 change in stage 3	0.161	3.239	<0.001
P change in stage 2	0.139	2.942	<0.001
P change in stage 3	0.090	1.897	0.063
**CHARACTERISTICS**	**Rate of Transferable Embryos**		**P**
	**B**	**t**	
E2 change in stage 2	-0.071	-1.396	0.104
E2 change in stage 3	-0.150	-2.938	0.003
P change in stage 2	-0.083	-1.715	0.129
P change in stage 3	-0.008	-0.158	0.836

## DISCUSSION

Our analysis demonstrates that, across all phases of the cycle, fluctuations in serum E2 concentrations were consistently more pronounced than those of P. E2 levels increased progressively and significantly at each stage, with the most marked surge occurring immediately prior to hCG administration. This trajectory aligns with prior studies (Hariton *et al*., 2017; McEvoy *et al*., 2022), which identify the late follicular peak in serum estradiol as a critical indicator of follicular maturation (Persani *et al*., 2014). The association between serum estradiol levels and oocyte retrieval rates in assisted reproductive technologies has been well-documented (Cedars, 2022; Fatemi *et al*., 2021; He *et al*., 2023). We observed that a sharp rise in serum E2 levels during the final three days of COS was predictive of a higher oocyte yield. This finding is consistent with earlier research showing a positive correlation between steroid hormone levels during superovulation and the number of mature follicles (Fiers *et al*., 2017; Li *et al*., 2020)-factors influenced by ovarian reserve and responsiveness (Amaral *et al*., 2019). However, the simultaneous decline in embryo quality may reflect the adverse effects of supraphysiologic hormone elevations. Specifically, an abrupt rise in estradiol during the late follicular phase may impair follicular development, compromise oocyte competence, and reduce embryonic developmental potential post-fertilization (Levin & Rottenstreich, 2019; Li *et al*., 2019; Muñoz *et al*., 2012).

Several factors appear to influence the late-stage rise in estradiol, including the underlying cause of infertility, AFC, AMH levels, BMI, ovarian volume at various stages, stimulation duration, total gonadotropin dose, and initial dosage (Bellver *et al*., 2022; Višnová *et al*., 2021; Schneider *et al*., 2024). Notably, a favorable pattern of follicular growth in earlier stages generally predicts a similar upward trend in the final three days. Future studies should investigate the relationship between dosage modifications and the dynamic changes in serum estradiol levels during COS.

In our cohort, lower starting doses and shorter stimulation durations emerged as independent protective factors associated with a more rapid increase in estradiol levels during the final three days. This may reflect an inverse relationship between ovarian responsiveness and gonadotropin requirements. Nevertheless, these findings warrant validation through prospective studies and further stratification by infertility etiology, in order to address potential biases arising from methodological limitations.

Collectively, our results suggest that estradiol elevation serves as a more sensitive biomarker of follicular development than progesterone. The E2 surge is instrumental in promoting oocyte maturation-a pivotal determinant of successful in vitro fertilization. Accordingly, it is essential to define the optimal range of estradiol increase during the final days of COS, particularly in patients undergoing long protocols initiated in the early follicular phase. Tailored COS strategies may help optimize both oocyte yield and quality, mitigate the negative impact of late-phase estradiol fluctuations on embryo competence, and enhance clinical outcomes through careful hormonal monitoring, timely medication adjustments, and precise timing of ovulatory trigger administration.

However, this single-center retrospective study is subject to inherent limitations, including potential selection bias and residual confounding. Additionally, heterogeneity in infertility etiology was not fully controlled. These findings warrant confirmation through well-designed prospective, multi-center studies.

## CONCLUSION

Monitoring E2 dynamics, particularly during the final days of COS, provides valuable insight into follicular development and stimulation response. High late-phase E2 levels predict greater oocyte yield but may reduce embryo quality. Individualized stimulation based on patient characteristics and hormone response is essential to optimizing IVF outcomes. Further research is needed to define optimal hormone trajectories to balance embryo quality and quantity.
